# A Rare Case of Hypothyroidism-Induced Rhabdomyolysis

**DOI:** 10.7759/cureus.37211

**Published:** 2023-04-06

**Authors:** Omar Z Syed, Khalid Ahmed, Ahmed Algohiny, Elmkdad Mohammed, Peter A Iskander, Douglas Klamp, Simin Nasr

**Affiliations:** 1 Internal Medicine, The Wright Center for Graduate Medical Education, Scranton, USA; 2 Family Medicine, The Wright Center for Graduate Medical Education, Scranton, USA

**Keywords:** general internal medicine, autoimmune disease, creatinine kinase, hypothyroidism, rhabdomyolysis

## Abstract

Rhabdomyolysis is a condition caused by muscle breakdown. It can be usually associated with pain, weakness, and elevated creatinine kinase levels on laboratory testing. There are various triggers, some of which can include trauma, dehydration, infections, and, as in this case, autoimmune disorders. Here, we present a case of a patient with worsening muscle pain who was found to have elevated creatinine kinase levels and undiagnosed hypothyroidism, with symptoms improving with intravenous hydration and thyroid supplementation.

## Introduction

Muscle involvement is a common manifestation among patients with hypothyroidism, occurring in over 70% of cases [1]. Symptoms may include muscle pain and weakness, with laboratory findings indicating elevated creatine kinase (CK) levels [2]. In severe cases, patients may develop rhabdomyolysis, a serious medical condition in which muscle tissue breaks down and releases its contents into the bloodstream [3]. While rhabdomyolysis can be caused by various pathologies, including hypothyroidism, this association is considered rare [4]. When thyroid hormone levels are low, the body may not properly regulate muscle tissue breakdown. Hypothyroidism can be attributed to medication non-adherence, suboptimal dosing, or other undiagnosed diseases. Additionally, factors such as dehydration, low electrolyte levels, and medications, such as statins, can contribute to rhabdomyolysis in patients with hypothyroidism [5].

## Case presentation

A 34-year-old female with a past medical history of hypothyroidism presented to the hospital secondary to a two-week episode of worsening fatigue and generalized muscle cramps. The patient noted that she was previously on thyroid supplementation (although she could not recall the dosing) but had stopped a few years prior. This was secondary to a lack of establishing care with a primary care doctor after moving from a different state. Initial laboratory investigations revealed a white blood cell count of 5,000/μL, normocytic anemia with a hemoglobin level of 11.3 g/dL and hematocrit of 34.1%, and a normal platelet count of 147,000/μL. The mean corpuscular volume was 89 fL and red cell distribution width was 21.4%. Complete blood count and iron panel results were consistent with anemia of chronic disease, while serum folate and vitamin B12 levels were within normal limits. Her serum sodium level was 133 mmol/L, serum potassium level was 3.6 mmol/L, serum albumin level was 2.0 g/dL, serum alkaline phosphatase level was 91 U/L, serum alanine transaminase level was 179 U/L (normal = 4-36 U/L), serum aspartate transaminase level was 631 U/L (normal = 8-33 U/L), direct bilirubin level was 0.60 mg/dL, CK level was 20,507 U/L (normal = 30-145 U/L for females), and total bilirubin level was 2.1 mg/dL. Coagulation studies revealed a prolonged prothrombin time of 21.4 seconds, a normal partial thromboplastin time of 32.5 seconds, and an elevated international normalized ratio of 2.06. Respiratory panel polymerase chain reaction was positive for adenovirus, while urinalysis showed 3+ blood. The urine drug screen was positive for cannabinoids, and a chest X-ray was unremarkable for any acute cardiopulmonary disease (Table 1).

**Table 1:** Patient’s lab values on admission with reference ranges.

Laboratory test	Reference range	Result
White blood cell count	4.5–11 K/μL	5 K/μL
Hemoglobin	12.0–15.5 g/dL	11.3 g/dL
Hematocrit	35–45%	34.1%
Platelet count	150–450 K/μL	147 K/μL
Mean corpuscular volume	80–100 fL	89 fL
Red cell distribution width	11.5–14.5%	21.4%
Serum sodium level	135–145 mmol/L	133 mmol/L
Serum potassium level	3.5–5.0 mmol/L	3.6 mmol/L
Serum albumin level	3.5–5.0 g/dL	2.0 g/dL
Serum alkaline phosphatase	44–147 U/L	91 U/L
Serum alanine transaminase	4–36 U/L	179 U/L
Serum aspartate transaminase	8–33 U/L	631 U/L
Direct bilirubin	0.0–0.4 mg/dL	0.60 mg/dL
Creatine kinase	30–135 U/L	20,507 U/L
Total bilirubin	0.1–1.2 mg/dL	2.1 mg/dL
Prothrombin time	11–13 seconds	21.4 seconds
Partial thromboplastin time	25–35 seconds	32.5 seconds
International normalized ratio	0.8–1.2	2.06
Respiratory panel polymerase chain reaction	Not applicable	Positive for adenovirus
Urinalysis	Negative	3+ blood
Urine drug screen	Not applicable	Positive for cannabinoids
Thyroid-stimulating hormone	0.45–4.5 μIU/mL	45 μIU/mL
Free thyroxine	0.8–1.8 ng/dL	Low
Thyroid peroxidase antibody	<9 IU/mL	Elevated

The patient was admitted for further management and evaluation of rhabdomyolysis and elevated liver enzymes and was started on intravenous fluids. Physical and occupational therapy were consulted, and symptomatic management for respiratory symptoms was initiated. Gastroenterology was also consulted for transaminitis. On further questioning, the patient denied the use of acetaminophen-containing products, other over-the-counter medications, or supplements. She reported having two mixed drinks per week with her friends but denied alcohol abuse. She also denied a history of intravenous drug use. She denied a history of hepatitis.

Initial differential diagnosis included rhabdomyolysis with elevated liver enzymes, possibly due to a viral infection or autoimmune myositis with liver involvement. The patient was counseled on complete alcohol cessation. Viral hepatitis panel testing was performed which was negative for hepatitis A, B, or C infection. A liver ultrasound showed evidence of fatty liver. On further questioning, the patient reported that her muscle weakness was associated with difficulty standing up from a sitting position. Antinuclear antibody testing was positive, and rheumatology was consulted for possible muscle biopsy. Myositis-associated antibodies, including anti-Jo 1, anti-Ro, anti-La/SSM, anti-SM, and anti-RNP, were negative.

**Figure 1:**
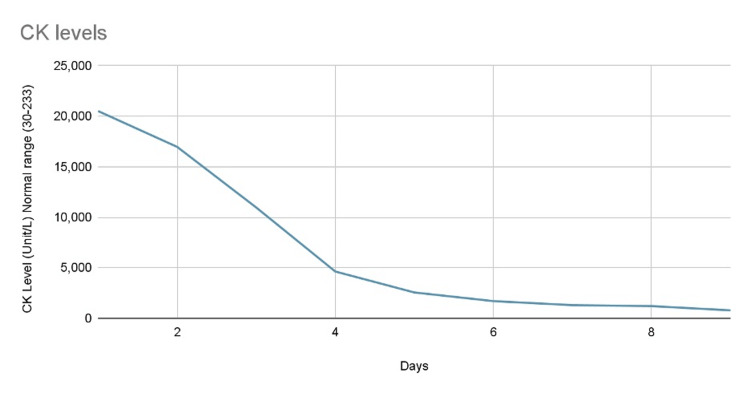
Graph depicting the downtrending of CK levels over the patient’s hospital course. CK: creatine kinase

The patient was continued on intravenous fluids to maintain hydration and electrolyte balance. CK levels were monitored and showed a decreasing trend, which was indicative of the resolution of rhabdomyolysis (Figure 1). Liver function tests (LFTs) were also noted to be decreasing over time.

Further history revealed that the patient had a remote history of thyroid disorder for which she was on thyroid replacement therapy in the past. Thyroid-stimulating hormone (TSH) was ordered accordingly, which was elevated at 43 with low free thyroxine (FT4) levels. The thyroid peroxidase antibody was also elevated at 26 IU/mL (normal <9 IU/mL), which was positive.

The patient was suspected to have rhabdomyolysis and myopathy secondary to untreated hypothyroidism. She was started on oral levothyroxine therapy. Before discharge, CK levels and LFTs had normalized, and the patient’s symptoms had significantly improved. She was discharged home with physical therapy arrangements. A repeat thyroid function test performed six weeks after discharge showed a normal TSH level, indicating an adequate response to treatment with levothyroxine.

## Discussion

Hypothyroidism is characterized by a wide range of symptoms, none of which are specific to the condition. Diagnosis typically relies on TSH and T4 measurements [6]. The majority of patients with overt hypothyroidism report a variety of muscular symptoms [7]. Although rare, hypothyroidism can present solely as rhabdomyolysis [4,8]. This can be characterized as a condition characterized by skeletal muscle necrosis and the release of intracellular muscle content into the bloodstream [9]. Strenuous exercise, trauma, electrolyte imbalance, infection, and certain drugs (such as statins and alcohol) are well-known triggers of rhabdomyolysis [10]. In the literature, only a few cases of rhabdomyolysis caused by hypothyroidism have been reported [11].

Hypothyroidism can lead to myolysis due to alterations in muscle fibers, including a shift from fast-twitch type II fibers to slow-twitch type I fibers, deposition of glycosaminoglycan, decreased contractility of actin-myosin units, reduced myosin ATPase activity, and decreased ATP turnover in skeletal muscles [12]. Inhibition of mitochondrial oxidative phosphorylation is the likely culprit behind these changes, which leads to decreased ATP production and altered metabolic pathways, including the Krebs cycle, glycogenolysis, and fatty acid degradation [13]. All of these changes can result in myopathy in patients with hypothyroidism, and in some cases, rhabdomyolysis may also develop.

The diagnosis of rhabdomyolysis is typically based on the presence of muscular symptoms (i.e., pain and weakness) and a fivefold increase in CK levels above the upper limit of normal values. Moreover, myoglobinuria may also be present [14]. In one case presented by Baghi et al. [15], a patient with TSH levels greater than 100 mIU/L presented with CK levels of 21,644 U/L. These levels began to gradually improve upon administration of levothyroxine therapy. Rhabdomyolysis induced by hypothyroidism typically results in a CK level that is less than 10 times the normal value, but in some cases, it may be elevated up to 70 times the normal value [16]. In patients with no apparent cause of rhabdomyolysis, thyroid function tests should be performed, and precipitating factors such as statin use, strenuous exercise, and a remote history of thyroid abnormalities should be carefully evaluated [17,18].

Initially, no apparent cause could be identified for the elevated CK levels in our patient. The patient denied any history of statin use or other medications commonly associated with myopathies. Therefore, hypothyroidism was considered a possible differential diagnosis and confirmed by laboratory evaluation. According to the current American Thyroid Association guidelines, the presence of elevated CK or lactate dehydrogenase levels for at least two weeks warrants testing for thyroid abnormalities [19]. Furthermore, it is important to pursue further investigation for potential coexisting autoimmune conditions related to hypothyroidism, such as Addison’s disease, vitiligo, pernicious anemia, and type 1 diabetes mellitus with autoimmune hypothyroidism.

The primary treatment for rhabdomyolysis is aggressive fluid repletion, although the optimal type and rate of fluid administration remain unclear. In general, 100-200 mL/hour of an isotonic solution is recommended to maintain renal perfusion. Serial CK measurements should be obtained until levels stabilize and stop rising. Levels below 5,000 U/L do not require intravenous fluids as they have a low risk of acute kidney injury [20]. In addition, TSH and FT4 levels should be measured every four to six weeks to assess the response to treatment, and the dose should be adjusted by 25-50 µg/day to achieve an appropriate response.

## Conclusions

Considering the wide variety in the causes of rhabdomyolysis, we suggest that hypothyroidism be included in the differential diagnosis, and physicians should not hesitate to perform thyroid function tests. Our case highlights the significance of measuring thyroid function in patients with rhabdomyolysis in the absence of hypothyroid symptoms or other causes of rhabdomyolysis. This approach can ensure a prompt diagnosis and treatment for such patients to help prevent delays in care.
